# Multi-omics analysis reveals the impact of influenza a virus host adaptation on immune signatures in pig tracheal tissue

**DOI:** 10.3389/fimmu.2024.1432743

**Published:** 2024-08-16

**Authors:** Helena Aagaard Laybourn, Chrysillis Hellemann Polhaus, Charlotte Kristensen, Betina Lyngfeldt Henriksen, Yaolei Zhang, Louise Brogaard, Cathrine Agnete Larsen, Ramona Trebbien, Lars Erik Larsen, Konstantinos Kalogeropoulos, Ulrich auf dem Keller, Kerstin Skovgaard

**Affiliations:** ^1^ Department of Biotechnology and Biomedicine, Technical University of Denmark, Kongens Lyngby, Denmark; ^2^ Department of Veterinary and Animal Sciences, University of Copenhagen, Copenhagen, Denmark; ^3^ Qingdao Key Laboratory of Marine Genomics, BGI-Qingdao, Qingdao, China; ^4^ Department of Virus and Microbiological Special Diagnostics, Statens Serum Institut, Copenhagen, Denmark

**Keywords:** influenza A virus, RNA-Seq, global proteomics, immune regulation, host metabolism, host adaptation

## Abstract

**Introduction:**

Influenza A virus (IAV) infection is a global respiratory disease, which annually leads to 3-5 million cases of severe illness, resulting in 290,000-650,000 deaths. Additionally, during the past century, four global IAV pandemics have claimed millions of human lives. The epithelial lining of the trachea plays a vital role during IAV infection, both as point of viral entry and replication as well as in the antiviral immune response. Tracheal tissue is generally inaccessible from human patients, which makes animal models crucial for the study of the tracheal host immune response.

**Method:**

In this study, pigs were inoculated with swine- or human-adapted H1N1 IAV to gain insight into how host adaptation of IAV shapes the innate immune response during infection. In-depth multi-omics analysis (global proteomics and RNA sequencing) of the host response in upper and lower tracheal tissue was conducted, and results were validated by microfluidic qPCR. Additionally, a subset of samples was selected for histopathological examination.

**Results:**

A classical innate antiviral immune response was induced in both upper and lower trachea after infection with either swine- or human-adapted IAV with upregulation of genes and higher abundance of proteins associated with viral infection and recognition, accompanied by a significant induction of interferon stimulated genes with corresponding higher proteins concentrations. Infection with the swine-adapted virus induced a much stronger immune response compared to infection with a human-adapted IAV strain in the lower trachea, which could be a consequence of a higher viral load and a higher degree of inflammation.

**Discussion:**

Central components of the JAK-STAT pathway, apoptosis, pyrimidine metabolism, and the cytoskeleton were significantly altered depending on infection with swine- or human-adapted virus and might be relevant mechanisms in relation to antiviral immunity against putative zoonotic IAV. Based on our findings, we hypothesize that during host adaptation, IAV evolve to modulate important host cell elements to favor viral infectivity and replication.

## 1 Introduction

Influenza A virus (IAV) is a zoonotic virus which can infect a wide range of avian and mammalian species (1–4). IAV causes respiratory illness worldwide leading to annual seasonal epidemics estimated to result in 3–5 million cases of severe illness and 290,000–650,000 deaths each year (5). IAV likewise gives rise to global pandemics, of which four have occurred over the past century (6, 7). The emergence of IAV pandemics is attributed to zoonotic transmission from avian or swine sources, followed by reassortment events (8, 9). This sequence of events leads to the ongoing emergence of new IAV strains capable of causing pandemics after adaptation to the new host. In order to understand the underlying molecular mechanisms behind zoonotic events, we need a better understanding of how the host reacts to both host-adapted IAV strains and non-adapted IAV strains. Innate immune responses are highly relevant when studying the zoonotic potential of IAV, as IAV must circumvent these innate mechanisms to successfully establish itself in a new host, which includes hijacking the metabolic machinery of its host cell in order to produce all necessary components for efficient viral propagation (10, 11).

During IAV infection, the antiviral innate immune response is activated through pattern-recognition receptors (PRRs). Upon recognition by PRRs, production of interferons (IFNs), pro-inflammatory cytokines, and chemokines is initiated. In general, type I and III IFNs stimulate the production of interferon stimulated genes (ISGs), which interfere with and restrict viral replication (12–14). The tracheal epithelium is crucial during respiratory viral infections as it serves as one of the initial sites of viral entry, where the virus adheres to and infects epithelial cells. Animal models are essential to study disease severity and viral replication in the affected tissues, given the scarcity of clinical respiratory tissue samples from human patients. Data on host immune responses in tracheal tissue after IAV infection is scarce and has mainly been studied *in vitro* or *in vivo* using small animal models (15–20) or chickens (21–23). Given the limited data obtained exclusively from smaller animal models, it would be pertinent to conduct such investigations in the highly translational pig model, which much more accurately mirrors human respiratory architecture and composition of the innate immune system (24).

In the present study, we performed an in-depth multi-omics analysis of the response in swine tracheal tissues to either swine- or human-adapted IAV three days after inoculation. This allowed us to obtain comprehensive insight into the dynamics of the antiviral immune response and host factors involved in the host cell environment, which play a role in viral replication efficiencies. It likewise provided insights to a better understanding of the mechanisms behind host adaptation and cross-species transmission.

## 2 Materials and methods

### 2.1 Preparation of virus inoculum

A swine-adapted virus, A/Swine/Denmark/2017_10298/4_4p1/2017 (H1N1) (swH1N1; accession no. MT666901-MT666908) and a human-adapted virus, A/Denmark/238/2020 (H1N1) (huH1N1; accession no. OQ062647-OQ062654) were propagated and passaged three times in Madin–Darby canine kidney (MDCK) cells. The viruses were stored at -80°C before inoculation (25). The titers were determined by tissue culture infectious dose 50% (TCID_50_) assay in MDCK cells, and diluted in Eagle’s Minimum Essential Medium (Gibco) to a TCID_50_/ml of 10^7^.

### 2.2 Experimental design

The experimental set-up has previously been described in details (25). Briefly, 22, confirmed IAV negative, seven-week-old Danish Landrace Crossbred pigs were included. The pigs were allocated into three groups and housed in separate isolation units. They were acclimatized for one week, fed non-pelleted feed, and had *ad libitum* access to water. Group 1 (control) consisted of six pigs, while group 2 and 3 included eight pigs each. The pigs were sedated before inoculation. A MAD Nasal intranasal mucosal atomization devie (Teleflex) was used for inoculation, as it delivers a fine mist of droplets in the range of 30 to 100 microns to more realistically mimic viral challenge. Group 1 was mock inoculated intranasally in one nostril by the MAD nasal device (Teleflex) containing 3 mL culture medium only, while group 2 and 3 were inoculated with 3 ml of 10^7^ TCID_50_/ml of the swine-adapted H1N1 strain, A/Swine/Denmark/3974/2017 (swH1N1), or the human-adapted H1N1 strain, A/Denmark/238/2020 (huH1N1) (25), respectively. The pigs were euthanized 3 days post inoculation, and tracheal tissue was collected and stored in either RNA*later* (Thermo Fisher Scientific) at -20°C until RNA extraction or washed in PBS, snap frozen in liquid nitrogen and stored at -80°C for proteomics analysis. Lung tissues for viral titration were stored in Eppendorf tubes at -80°C.

The animal experiment was performed under biosafety level 2 conditions and under an animal study protocol approved by The Danish Animal Experimentation Council (protocol no. 2020–15-0201–00502).

### 2.3 Proteomics workflow

#### 2.3.1 Sample preparation

The mucosal membrane of upper and lower trachea was stripped from the cartilage and homogenized in lysis buffer 1 (6 M guanidine hydrochloride (GuHCl), 10 mM tris(2-carboxyethyl)phosphine (TCEP), 40 mM chloroacetamide (CAA), 50 mM 4-(2-hydroxyethyl)-1-piperazineethanesulfonic acid (HEPES), pH 8.5, with cOmplete™ Mini EDTA-free protease inhibitor cocktail) using a TissueLyser II (Agilent) and one steel bead twice for one minute at 1–30 Hz. The samples were incubated for 5 min at 95°C for reduction and alkylation of cysteines. To remove contaminants, lysates were precipitated in acetone overnight at -20°C, centrifuged for 20 min at 4000x g at 4°C, and resuspended in lysis buffer 2 (6 M GuHCl, 50 mM HEPES, pH 8.5, with cOmplete™ Mini EDTA-free protease inhibitor cocktail). Protein concentration was determined with NanoDrop One (Thermo Fischer Scientific). A total of 50 µg of extracted protein from each sample were digested with LysC (TriChem) (1:100 protease to protein (w:w)) at 37°C for 4 hours at 300 rpm after adjusting the GuHCl concentration to 0.5 M with digestion buffer (10% acetonitrile, 50mM HEPES, pH 8.5), followed by an overnight digestion at 37°C with trypsin (Promega) (1:100 protease to protein (w:w)). The digestion was stopped by adding 20% trifluoroacetic acid (TFA) to all samples (1% final concentration). Samples were desalted using a Solaµ HRP 96 well plate (Thermo Scientific) by centrifugation at 1500 rpm for 1 minute in every step. The plate columns were activated by 100% methanol (Merck), buffer B (40% acetonitrile, 0.1% formic acid) and buffer A’ (3% acetonitrile, 1% TFA). The samples were loaded on to the plate, followed by washing with buffer A (0.1% formic acid) and elution using buffer B. All of the samples were dried by vacuum at 45°C until dry. For spectral library generation, pools including control and infected samples were prepared for digestion with a final protein concentration of 200 µg for each pool. The pooled samples were digested and desalted as described for each individual sample. The pooled samples were fractionated into 10 fractions using high pH fractionation and a Thermo Acclaim PA2 (3 um, 300 um x 150 nm) column, using a Dionex Ultimate 3000 system (U3000). The samples ran at 5 µl/min at approximately 400 bar, using AMBIC buffer (5mM ammonium bicarbonate) and buffer B (100% acetonitrile).

Individual samples and library samples, corresponding to 500 ng of peptides, were placed on Evotips that had been activated by 100% acetonitrile, 100% isopropanol and buffer A according to the manufacturer loading protocol. For liquid chromatography/mass spectrometry (LC-MS) analysis, an Evosep One HPLC system was used in-line with an Orbitrap Exploris 480 mass spectrometer (Thermo Fischer Scientific). The instrument was operated in positive polarity in data independent acquisition (DIA) mode, with a gradient of 118 minutes (10SPD) using a Whisper column (EV1112, 15 cm length, 75 µm diameter, and bead size 1.9 µm) running at 100 nl/min, with column heating at 30°C, and a transfer tube temperature of 240°C. MS scans were acquired in between the MS/MS DIA scans (26) for precursor quantification. The global parameters were set to nano-spray ionization, with a static positive ion voltage of 2000V and 600V for the negative ion.

The resolution of Orbitrap for MS scan acquisition was set to 120,000 with FAIMS ON with –45 CV at standard resolution mode. The resolution for the DIA scans was 60,000. The RF lens was 40% for both MS and MS/MS DIA scans. Normalized AGC Target of 300% was applied for the MS scans and the maximum injection time was automatic. The normalized AGC Target was 1,000% for the MS/MS DIA scans and the maximum injection time was automatic. The scan range for the MS scans was 400–1000 m/z. The scan range for the MS/MS DIA scans was 400–600 m/z, 600–800 m/z and 800–1000 m/z. The HCD collision energy was 32%. The window size of DIA scan was 6 m/z, with an overlap of 1 m/z.

#### 2.3.2 Spectral library generation

The spectral library was generated from DIA search archives using Spectronaut (27) (17.4.230317.55965, Biognosys AG), using default settings except the digestion type, which was set to Trypsin/P and LysC. Modifications used for the search were fixed carbamidomethylation of cysteines (C, +57.021464 Da), and variable acetylation (protein N-terminus, +42.0106 Da), oxidation (M, +15.995 Da), and deamidation (N, +0.984 Da). The protein sequences used to generate the spectral library were *Sus scrofa* proteome (UniProt fasta, access date: 2022–04, 48349 entries containing both reviewed and un-reviewed TrEMBL sequences for taxID 9823), and two in-house files generated from the sequenced viral strains (25) for both the human-adapted IAV (huH1N1) and swine-adapted IAV (swH1N1). The library comprised a total of 93,131 peptides and 10,003 proteins.

#### 2.3.3 DIA data analysis

The data analysis was performed using Spectronaut (17.4.230317.55965, Biognosys AG). The acquired spectra were searched against the spectral library generated in Spectronaut. Searches were run using Precursor PEP cutoff of 0.1, Protein Qvalue cutoff (run) of 0.05, and Protein PEP cutoff of 0.5. Quantification was performed using Spectronaut’s version of MaxLFQ (28) algorithm with precursor quantification on MS level. Cross-run normalization was performed with default settings, while no imputation method was used.

### 2.4 Transcriptomics workflow

#### 2.4.1 RNA extraction

Whole sections of lower trachea were homogenized in QIAzol Lysis reagent (QIAGEN) using gentleMACS M tubes (Miltenyi Biotec). Total RNA was extracted using the miRNeasy mini kit (QIAGEN) and treated with an RNase-free DNase set (QIAGEN) according to the manufacturer’s instructions. RNA quantity and purity were estimated using the NanoDrop One (Thermo Fisher Scientific) and the RNA integrity number was assessed by the Agilent RNA 6000 Nano Kit on the Bioanalyzer 2100 system (**Supplementary Table S1**) (Agilent Technologies).

#### 2.4.2 Library generation and sequencing

RNA-sequencing (RNA-seq) libraries were constructed with a non-stranded and PolyA selection method according to the manufacturer’s specifications (BGI Genomics). Paired-end reads with a length of 100 bp were sequenced using the DNBSEQ platform. The raw reads were filtered, which included adaptor removal, removal of contamination, and low-quality reads (ambiguous bases (N) higher than 5%, low-quality bases (quality score less than 10) higher than 20%, and having sequencing adaptor contamination).

#### 2.4.3 RNA-seq data analysis

After filtering, clean reads were mapped to the pig genome (Sscrofa11.1, NCBI accession: GCA_000003025.6) using HISAT2 (29). RSEM (30) was used to annotate transcripts to prepare a transcript reference for subsequent calculation of gene expression levels. The clean reads were mapped to the transcript reference to quantify the gene expression using Bowtie2 (31). RSEM (30) was used to estimate the gene expression levels based on fragments per kilobase of exon per million fragments mapped (FPKM) for subsequent analysis of the differentially expressed genes (DEGs). Data was normalized and DEGs were identified between different comparison groups by DESeq2 (32).

#### 2.4.4 Validation of RNA-seq data by high-throughput microfluidic qPCR

A subset of 93 genes (including six potential reference genes for data normalization) with annotations from a statistical analysis of the RNA-seq was selected for validation using high-throughput reverse transcription quantitative real-time polymerase chain reaction (RT-qPCR). Primers for the selected genes are shown in **Supplementary Table S2**. Primer design, cDNA synthesis, and cDNA pre-amplification were performed as described before (“Man submitted”, Laybourn HA, Pedersen AG, Brogaard B, Polhaus CH, Kristensen C, Trebbien R, et al.). High-throughput qPCR was carried out using 96.96 Dynamic Array IFC chips (Standard BioTools Inc.) on the BioMark real-time platform using a previously described PCR protocol (“Man submitted”, Laybourn HA, Pedersen AG, Brogaard B, Polhaus CH, Kristensen C, Trebbien R, et al.). Data was processed as described previously (“Man submitted”, Laybourn HA, Pedersen AG, Brogaard B, Polhaus CH, Kristensen C, Trebbien R, et al.). A biologically relevant cut-off value of log_2_ fold change (lfc) of ±1 compared to control was used. The change in gene expression level was considered statistically significant with a p-value below 0.05 (Student’s *t*-test).

### 2.5 Histopathology and IAV staining

Histological evaluations were performed on three pigs from the control group and two pigs from each inoculated group for both upper and lower trachea samples. Tissues were fixed in 10% neutral-buffered formalin for a week. Formalin-fixed tissue was embedded in paraffin wax and sliced into 2–3 µm sections. Sections were stained with hematoxylin and eosin (H&E). Immunohistochemical staining targeting IAV was performed as previously reported (33) by anti-influenza A (NP) antibody (diluted 1:100,000 in 1% BSA/Tris buffered saline) (HYB 340–05, SSI Antibodies) and UltraVision ONE HRP-Polymer (AH diagnostics). The staining was visualized by adding DAB substrate (Cell Marque). The sections were counterstained by Mayer’s hematoxylin (VWR). An isotype control (Agilent Technologies) (diluted in 1% BSA/Tris buffered saline to the same protein concentration as the anti-influenza A (NP) antibody) was used as a negative control.

### 2.6 Quantification and characterization of IAV

An in-house modified version of an RT-qPCR assay was used to detect IAV by targeting the matrix gene (M-gene) using the SensiFast Probe No-ROX One-Step Mix kit (Meridian Bioscience) as described before (25). Quantification was based on a 10-fold dilution series of the target sequence with known copy numbers. To determine the amount of infectious virus in the lungs, the lung specimen were homogenized, sterile filtered, and titrated in MDCK cells. The TCID_50_/ml was calculated using the Reed–Muench method (34).

A pairwise comparison of the amino acid sequences of the viral proteins of the inoculum strains was performed using CLC Main Workbench version 22.0 (QIAGEN) to measure the percentage identity. In addition, a sequence alignment of the NS1 sequences of the inoculum strains was performed.

### 2.7 Statistical analysis

The global proteomics data was analyzed using R studio (R version 4.2.2), the data was log_2_ transformed and checked for normality. A one-way ANOVA was performed, followed by a Tukey *post hoc* analysis to correct for multiple testing. In the RNA-seq data, the p-values of the DEGs were corrected for multiple testing using Benjamini-Hochberg false discovery rate (FDR) (35). Differentially expressed proteins (DEPs) and DEGs were defined with a p-adjusted value (padj) below 0.05 and with a lfc of ±1. DEGs and DEPs were functionally enriched by alignment to the Kyoto Encyclopedia of Genes and Genome (KEGG) (36) databases using enrichKEGG (37, 38).

## 3 Results

### 3.1 Global proteomics analysis highlights the importance of proteins associated with defense response to virus in the lower trachea upon IAV infection

**Figure 1:**
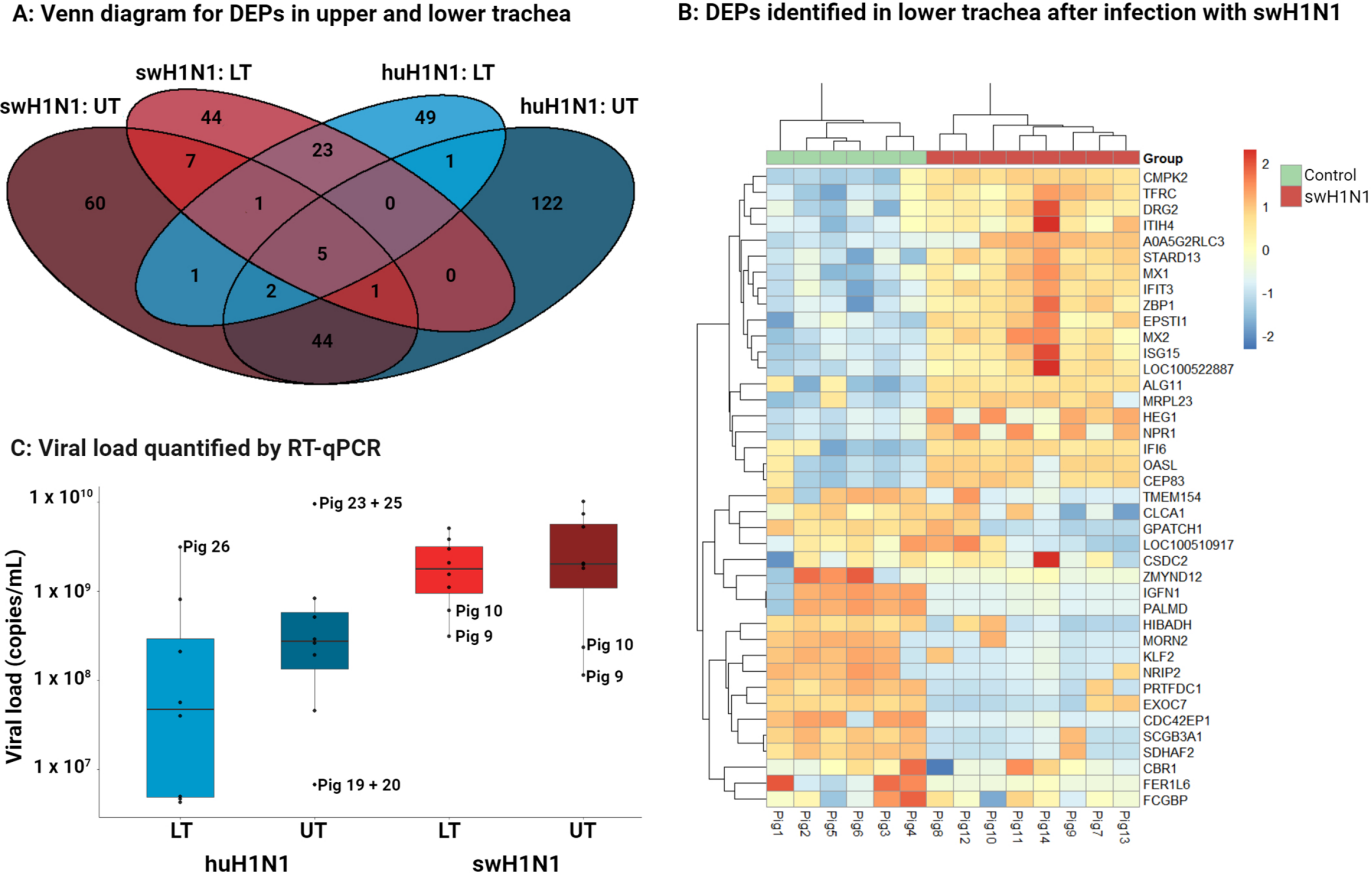
Diversity in DEPs and amount of viral RNA between upper trachea (UT) and lower trachea (LT). **(A)** Venn diagram illustrating the distribution of the significantly changed protein abundance for upper trachea (UT) and lower trachea (LT) after infection with the swine-adapted (swH1N1) or human-adapted (huH1N1) strain. **(B)** Top 20 significantly higher or lower abundant proteins (based on padj) in lower trachea after infection with swH1N1 (red) compared to control (green). The color key from blue to red indicates low to high protein expression, respectively. **(C)** Viral load quantified by RT-qPCR in each trachea sample (dots) from the two infected groups 3 days post inoculation (light blue, huH1N1 LT; dark blue, huH1N1 UT; light red, swH1N1 LT; dark red, swH1N1 UT), the box represents the interquartile range (IQR) with median indicated by the horizontal line and the whiskers extend to the data points that are 1.5 times IQR. All control pigs were IAV negative.

After global proteomics analysis of the upper and lower trachea, 8935 protein groups (76,690 peptides) were identified of which 3830 (17,526 peptides) were quantified due to the stringent search parameters used for quantification. All DEPs identified after either infection and their expression levels can be found in in **Supplementary Table S3**. Only five DEPs were common for both infections in both upper and lower trachea, including four interferon stimulated proteins (OASL, MX1, IFIT3, and ISG15) and the GTPase, DRG2. The limited overlap of DEPs between upper and lower trachea after either infections (**Figure 1A**) indicates a diverse protein expression between the two tissues and highlights the importance of investigating the tissues separately. Clustering of mock inoculated control pigs and pigs inoculated with either swH1N1 or huH1N1 showed that infected pigs clustered distinctly from control pigs (**Figure 1B**, **Supplementary Figure S1**). The highest viral loads were seen after infection with swH1N1 with a similar viral load in the upper (median of 2.03 x 10^9^ copies/ml) and lower trachea (median of 1.81 × 10^9^ copies/ml) (**Figure 1C**). After infection with huH1N1, the upper tracheal tissue exhibited higher viral load (median of 2.76 x 10^8^ copies/ml) than in lower trachea (median of 4.83 × 10^7^ copies/ml). Infectious virus in the lung was found in 8/8 pigs after infection with the swH1N1 (mean TCID_50_/ml of 2.18 x 10^5^) and 6/8 pigs after infection with huH1N1 (mean TCID_50_/ml of 6.15 x 10^4^) (25).

Several viral proteins were identified by proteomics after both infections, including M1, NS1, PB1, HA and NP. HA and NP were quantified after infection with swH1N1 in upper and lower trachea and both were highly abundant, which agrees with the viral load. No viral proteins could be quantified after infection with huH1N1 (**Supplementary Table S3**).

More DEPs were identified in the upper trachea compared to lower trachea after infection with either IAV strain (**Figure 1A**, **Supplementary Table S3**). Still, a larger percentage of the DEPs linked to defense response to virus (GO:0051607) was detected in the lower trachea following infection with either huH1N1 (9% of the DEPs) or swH1N1 (9% of the DEPs) compared to upper trachea (4% and 5%, respectively). Given that a greater percentage of DEPs linked to the antiviral immune response was detected in the lower trachea after infection with either huH1N1 or swH1N1, we decided to expand our omics data by conducting a transcriptional analysis of lower trachea using RNA-seq.

### 3.2 Multi-omics analysis revealed a classical antiviral immune response in lower trachea

Approximately 2 billion clean paired-end reads with a length of 100 bp were generated in the RNA-seq analysis of lower trachea. On average, 88.9 million (73.0–94.6 million) clean reads were obtained for each sample. Among the samples, 94.8–97.0% of the clean reads were mapped to the porcine reference genome. Moreover, 90.6–93.7% of the clean reads were uniquely mapped (mapped to exactly one location within the reference genome) **Supplementary Table S4**). All DEGs identified after either infection and their expression levels can be found in **Supplementary Table S5**.

**Figure 2:**
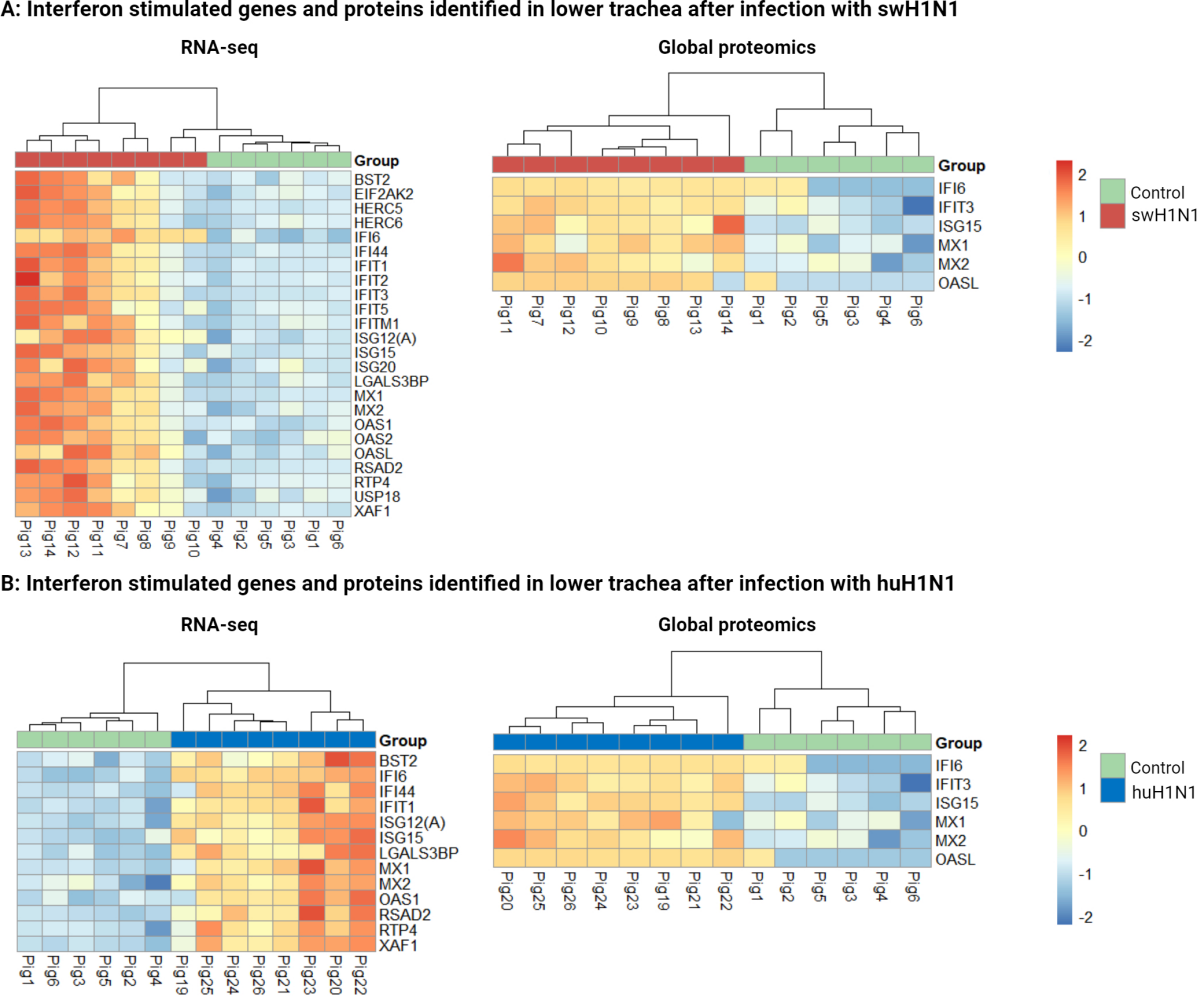
Interferon stimulated genes and proteins identified by RNA-seq and global proteomics in lower trachea after both infections. **(A)** Heatmaps showing all differentially expressed interferon stimulated genes (left) and proteins (right) in lower trachea comparing the control group (green) with the pigs infected with the swine-adapted strain (swH1N1) (red). **(B)** Heatmaps showing all differentially expressed interferon stimulated genes (left) and proteins (right) in lower trachea comparing the control group (green) with the pigs infected with the human-adapted strain (huH1N1) (blue). The color key from blue to red indicates low to high gene/protein expression, respectively.

A classical antiviral immune response was identified including high expression of interferon stimulated genes and proteins using multi-omics after infection with either swH1N1 and huH1N1 in the lower trachea (**Figure 2**). Four important antiviral ISGs, MX1, MX2, ISG15, and IFI6, were found to be upregulated after infection with either strains of IAV in the lower trachea (**Figure 2**) (**Supplementary Tables S3**, **S5**).

KEGG enrichment analysis was performed to identify the biological pathways associated with the DEGs and DEPs in lower trachea after infection with swH1N1 or huH1N1. Not surprisingly, most identified genes and proteins belonged to pathways involved in the antiviral immune response. Of the top 10 pathways enriched after infection with swH1N1 compared to control, most were related to response to RNA viruses (including SARS-CoV-2, IAV, and NOD signaling pathway) and cytokine signaling (Cytokine interactions, IL17 signaling, and TNF signaling) (**Supplementary Figure S2**). Likewise, enriched pathways after infection with huH1N1 compared to control were related to response to viruses (including SARS-CoV-2, IAV, Hepatitis C, and Epstein-Barr virus) and their recognition (RIG-I-like receptor signaling pathway and cytosolic DNA-sensing pathway) (**Supplementary Figure S3**).

The greater number of DEGs found after infection with swH1N1 compared to huH1N1 (**Supplementary Table S5**) might be connected with the higher viral load and the high abundance of viral proteins found after infection with swH1N1. Interestingly, the two pigs with the lowest viral load after infection with swH1N1 (pig 9 and 10) clustered together with the control pigs in the RNA-seq analysis (**Figure 2A**), but not in the global proteomics analysis (**Figures 1B**, **2A**).

### 3.3 Strong host transcription of a variety of interferon stimulated genes after IAV infection

After infection with huH1N1, a total of 28 DEGs were identified when compared to the controls. The 17 upregulated genes were all ISGs (**Figure 2B**) or involved in their activation (*DDX58* (RIG-I)*, IRF7*, and *DDX60*), except for *ANGPTL4* (**Supplementary Table S5**). *ANGPTL4* is involved in the regulation of glucose homeostasis and the lipid metabolism. Likewise, the most abundant group of upregulated genes after infection with swH1N1 was ISGs (**Figure 2A**) or genes involved in their activation (*DDX58* (RIG-I), *ZBP1, IFIH1* (MDA5), *IRF7*, and *DDX60*) (**Supplementary Table S5**). The 17 DEGs upregulated after infection with huH1N1 were shared with the swH1N1 infection (*DDX58* (RIG-I)*, RSAD2* (Viperin)*, MX2, MX1, OAS1, ANGPTL4, IRF7, ISG15, IFIT1, ISG12(A), DDX60, BST2, IFI44, XAF1, LGALS3BP, RTP4*, and *IFI6)*. In contrast, 244 genes (227 unique for this infection) were differentially expressed after infection with swH1N1 compared to control (**Supplementary Table S5**) and included a substantial number of ISGs (*HERC5, HERC6, IFIT2, IFIT3, IFIT5, IFITM1, ISG20, OAS2, OASL*, and *USP18*). Based on the RNA-seq data, *IFI6* was the most strongly upregulated gene after either infection (swH1N1: lfc = 2.27, padj = 1.48 x 10^-36^; huH1N1: lfc = 2.48, padj = 4.43 x 10^-44^). Genes encoding protein components of the cytoskeleton (*NEB*, *MYOM2*, *MYOZ1*, and *MYBPC1*) and surfactant proteins (A1, C, and D) were among the most significantly downregulated genes upon infection with swH1N1 (**Supplementary Table S5**).

**Figure 3:**
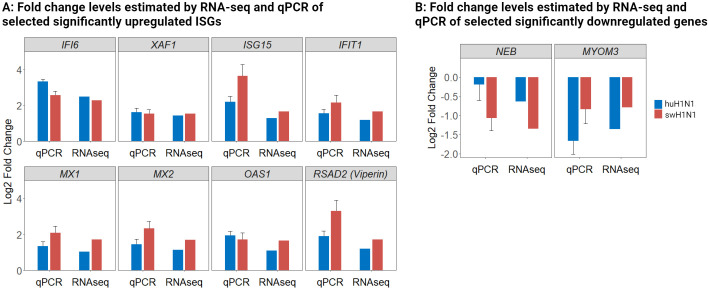
Validation of RNA-seq data with qPCR of both upregulated and downregulated genes. **(A)** Log_2_ fold change levels estimated by RNA-seq and qPCR of selected significantly upregulated antiviral genes in the IAV inoculated groups (huH1N1, blue; swH1N1, red.) relative to mock inoculated controls. **(B)** Log_2_ fold change levels estimated by RNA-seq and qPCR of selected significantly downregulated genes in the IAV inoculated groups (huH1N1, blue; swH1N1, red.) relative to mock inoculated controls. SEM is depicted by error bars.

To verify the results of the RNA-seq, a panel of the identified genes was studied by microfluidic high-throughput qPCR (**Supplementary Table S2**). In agreement with the RNA-seq results, a greater number of DEGs were identified after infection with swH1N1 compared to huH1N1 (**Supplementary Table S6**). The expression levels of both up- and downregulated genes were confirmed to be significantly regulated in the two infected groups compared to control by qPCR (p < 0.005) (**Figures 3A, B**). Expression levels were consistent with the RNA-seq results, though with the tendency of fold changes being of a slightly greater magnitude for some genes when investigated with qPCR (**Figure 3**, **Supplementary Table S6**).

### 3.4 Immune response to viruses with different host adaptation might involve central components of the JAK-STAT pathway and the pyrimidine metabolism

**Figure 4:**
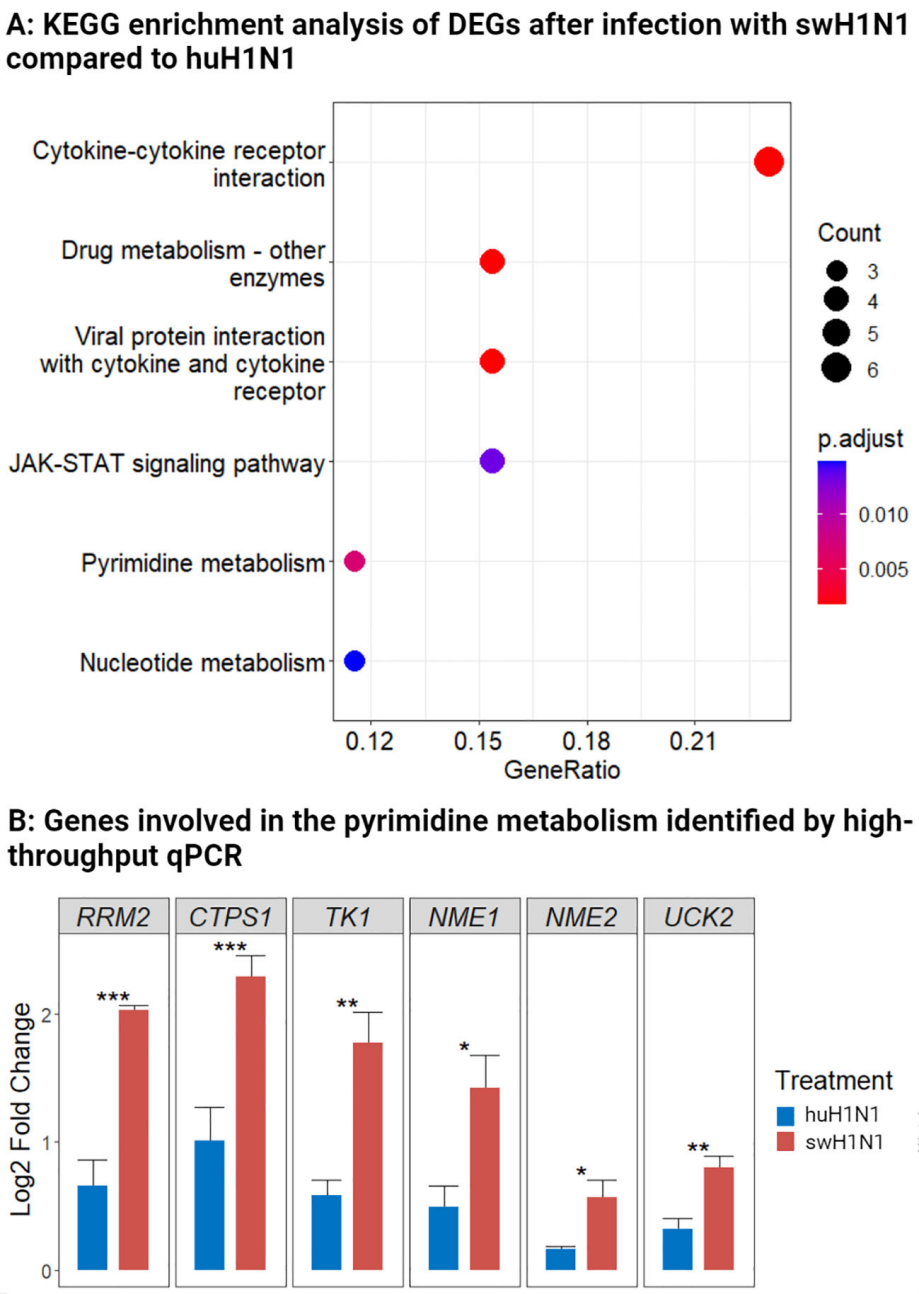
Components of the JAK-STAT pathway and the pyrimidine metabolism were regulated after infection with swine-adapted strain (swH1N1) compared to human-adapted strain (huH1N1). **(A)** Using KEGG enrichment analysis, we identified biological pathways associated with the DEGs comparing pigs inoculated with swH1N1 to those inoculated with huH1N1. The bubble diagram indicates the ratio of enriched DEGs to the total number of identified genes in a certain pathway. Circles indicate the number of genes in the corresponding pathway, and color depicts the adjusted p-value. **(B)** Log_2_ fold change levels of genes involved in the pyrimidine metabolism after infection with swH1N1 (red) and huH1N1 (blue). SEM is depicted by error bars. *p < 0.05, **p < 0.01, and ***p < 0.001 (Student’s *t*-test).

When comparing expression levels of proteins and genes in the lower trachea directly between the swH1N1 and huH1N1 inoculated pigs, 67 DEPs and 52 DEGs were identified (**Supplementary Table S3**, **S5**). Using KEGG enrichment analysis of all DEGs, we found a significant proportion of differentially expressed genes to be associated with the JAK-STAT signaling pathway (**Figure 4A**). The JAK-STAT pathway plays a pivotal role in orchestrating the host antiviral immune response by mediating the production of cytokines, chemokines, and interferons and activation of antiviral genes. Generally, infection with swH1N1 induced a more pronounced antiviral immune response with enriched genes associated with cytokine signaling (**Figure 4A**).

Differential expression of genes involved in the pyrimidine metabolism pathway was likewise enriched between the two infections (**Figure 4A**). *RRM2, CTPS1*, and *TK1* were highly expressed after infection with swH1N1 compared to huH1N1 (**Supplementary Table S5**). These results motivated a further investigation of genes associated with the pyrimidine metabolism during IAV infection by qPCR analysis. In agreement with the RNA-seq results, *RRM2*, *CTPS1*, and *TK1* were significantly expressed after infection with swH1N1 relative to the pigs infected with huH1N1 (**Figure 4B**). Furthermore, other genes related to the pyrimidine pathway, namely *NME1, NME2*, and *UCK2*, were significantly higher expressed after infection with the swH1N1 relative to the pigs infected with huH1N1, albeit occasionally with modest fold changes (**Figure 4B**). Furthermore, proteins related to the cytoskeleton and cytoskeleton remodeling (KRT2 and PLXNA4) and genes coding for components of the cytoskeleton, *KRT13* (LOC100515166)*, KRT17* (LOC100737113)*, KRT6A*, and *BFSP1*, were significantly higher expressed after infection with swH1N1 compared to huH1N1 (**Supplementary Table S3**, **S5**).

### 3.5 Necrotizing tracheitis was only identified after infection with swH1N1

**Figure 5:**
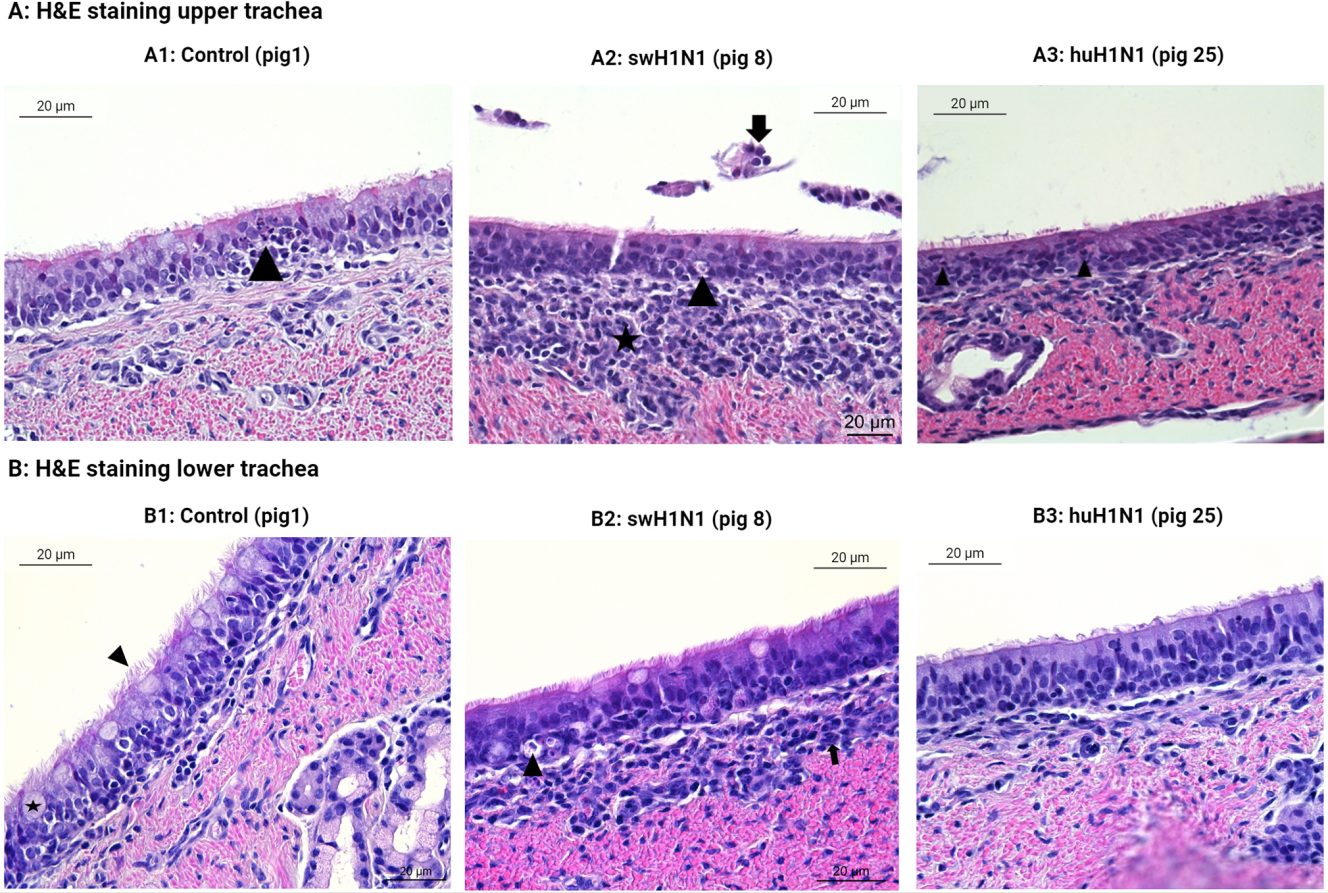
Histopatological changes in the upper **(A)** and lower **(B)** trachea after IAV infection. Representative images of the hematoxylin and eosin (H&E) staining of swine tracheal tissues from the control pigs (Pig 1, 3, and 5), the swine-adapted (swH1N1) infection (Pig 8 and 11), and the human-adapted (huH1N1) infection (Pig 22 and 25). (A1) Areas with moderate infiltration of neutrophils (arrowhead) in upper trachea in control pigs. (A2) Moderate infiltration of mononuclear cells in lamina propria (star), desquamated epithelial cells (arrow), and single cell necrosis of the tracheal epithelium (arrowhead) after infection with swH1N1 in upper trachea. (A3) Changes in huH1N1 infected pigs were comparable to control pig with areas with moderate infiltration of neutrophils (arrowheads). (B1) No changes were observed in the control pigs in lower trachea. Goblet cells (star) and cilia (arrowhead) are present. (B2) Single cell necrosis (arrowhead) of tracheal epithelial cells and mild infiltration of mononuclear cells in lamina propria (arrow) after infection with swH1N1 in the lower trachea. (B3) No changes were observed after infection with huH1N1 in the lower trachea.

Histopathological examination of the tracheal tissue sections showed necrosis and infiltration of immune cells after infection with swH1N1 in both examined animals in both the upper and lower trachea (**Figures 5A2, B2**, respectively).

In the lower trachea, lesions comparable to an acute, multifocal, mild to moderate, necrotizing tracheitis were observed in both pigs infected with swH1N1 (**Figure 5B2**), which could be linked to the host immune response. Apoptosis related proteins (ANXA5 and Caspase-13/CASP4) and *CAPN14, USP18, XAF1, Caspase-13/CASP4*, and *ISG12* transcripts were significantly upregulated after infection with swH1N1 compared to controls. The observed infiltration of immune cells after infection with swH1N1 agrees with the upregulation of chemotactic factors (*AMCF-II, CXCL8/IL8, CXCL2, CXCL10*, and *CCL20*) and their receptors (*CCR1* and *CXCR2*) at the transcriptional level (**Supplementary Table S5**). The lesions observed after infection with swH1N1 could be linked to the increased transcription of pro-inflammatory cytokines (*IL1A, IL1B, IL6, IL19, IL20*, and *IL27*) and other inflammatory genes from the S100 family (*S100A2* and *S100A8*). Histopathological examination of pigs infected with huH1N1 did not reveal infiltration of any immune cells or necrosis in the lower trachea (**Figure 5B3**). In agreement, no cytokines or chemokines were found to be significantly regulated at the transcriptional level (**Supplementary Table S5**) and two proteins related to recruitment and activation of immune cells were less abundant post infection compared to controls (CCL26 and IL13) (**Supplementary Table S3**). Consistent with the absence of necrosis, we identified a significant lower abundance of the pro-apoptotic protein PDCD10 in this group.

In the upper trachea, similar histopathological changes were identified as in lower trachea after infection with swH1N1 with one pig showing squamous metaplasia (**Figure 5**A2). Histological changes after infection with huH1N1 revealed changes comparable to control pigs or an acute, mild, suppurative tracheitis (**Figure 5A3**). However, no immune cell markers, chemotactic factors, inflammatory proteins or apoptotic related proteins were present in upper trachea at this point after either infection compared to control pigs. Though, DFFA, which is an inhibitor of caspase mediated apoptosis, was markedly decreased after both infections (swH1N1: lfc = -11.63, padj = 3.18 x 10^-2^; huH1N1: lfc = -13.90, padj = 1.00 x 10^-2^).

**Figure 6:**
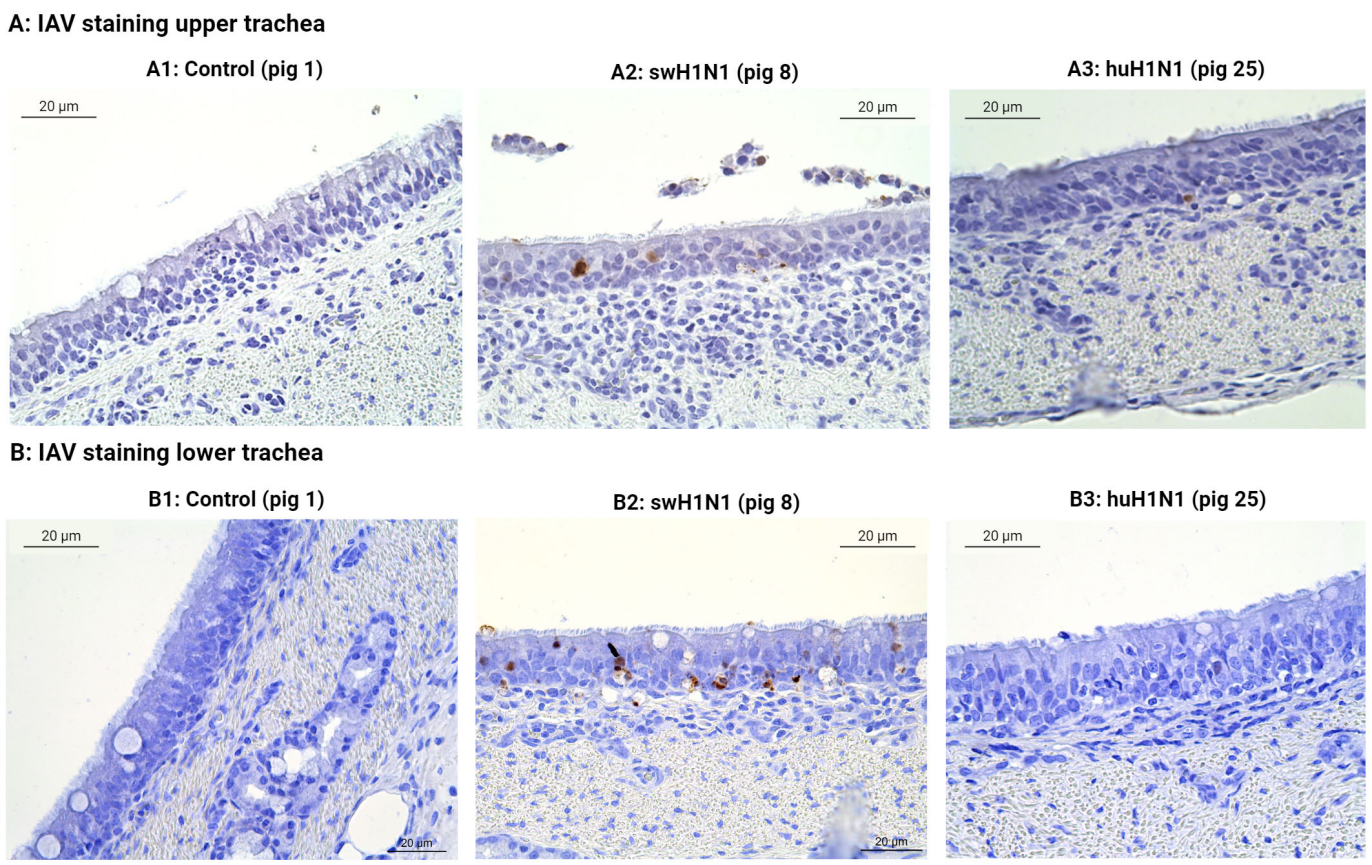
Immunohistochemical detection of IAV-positive cells in upper **(A)** and lower **(B)** trachea. The presence of IAV-positive cells (brown) was investigated by immunohistochemical staining of swine tracheal tissues from the control pigs (Pig 1, 3, and 5), the swine-adapted (swH1N1) infection (Pig 8 and 11), and the human-adapted (huH1N1) infection (Pig 22 and 25). (A1) No IAV-positive cells in the control group in upper trachea. (A2) IAV-positive cells (brown) were present in the epithelium after infection with swH1N1 in upper trachea. (A3) One IAV-positive cell (brown) was found in the epithelium after infection with huH1N1 in upper trachea. (B1) No IAV-positive cells in the control group in lower trachea. (B2) IAV-positive cells were present in the epithelium (brown) after infection with swH1N1 in lower trachea. (B3) No IAV-positive cells after infection with the huH1N1 in lower trachea.

The tracheal sections were also investigated for IAV-positive cells (**Figure 6**). No IAV-positive cells were found in the controls or in the pigs infected with huH1N1 for either tissue (except for one IAV-positive cell in the upper trachea), whereas several IAV-positive cells were found in the pigs infected with swH1N1 in both the upper and lower trachea (**Figures 6A2, B2**). This is consistent with viral nucleic acid measured by qPCR (**Figure 1B**) and the proteomics results, where HA and NP only could be quantified in the pigs infected with swH1N1 (**Supplementary Table S3**).

### 3.6 Influenza virus sequence analysis

A maximum-likelihood tree of the studied viruses and selected reference viruses have been created previously (25). Here, swH1N1 clusters within the swine H1N1pdm09 clade (1.A.3.3.2), whereas huH1N1 clusters with the human clade 6B. Pairwise comparisons of all viral gene sequences were performed resulting in high amino acid identities (>95%) between the two inoculum strains for most of the viral genes. The surface proteins, HA and NA, and the NS1 protein had a lower percent identity with 92.6% and 91.7% amino acid identity for HA and NA, respectively, while the NS1 protein, known for its role in immune evasion, had an amino acid identity of 94.5% between the two strains.

## 4 Discussion

Despite the involvement of tracheal epithelial cells in infection, replication, and spread of the IAV, there is currently limited information concerning the innate immune response of tracheal tissue to viral infection. By applying multi-omics analysis, we have elucidated host responses to IAV infection in tracheal tissue *in vivo* in swine, three days after experimentally infection with either swine-adapted (swH1N1) or human-adapted (huH1N1) IAV, and have provided results that possess significant translational potential for respiratory viral infections in this neglected tissue. By combining RNA-seq and MS-based proteomics, this study provides a comprehensive insight on multiple biological levels into mechanisms and defenses activated during infection with host-adapted IAV, as well as IAV adapted to infect and replicate in another mammalian host. The latter can be viewed as representative for early events that are likely to occur in a new host when IAV cross the species barrier during zoonotic and reverse zoonotic events.

A classical antiviral response was induced in the upper and lower trachea of pigs upon infection with both swH1N1 and huH1N1 compared to mock inoculated control pigs, primarily through activation of several ISGs and interferon stimulated proteins in the upper (OASL, MX1, IFIT3, and ISG15) and lower trachea (OASL, MX1, MX2, ISG15, IFIT3, IFI6, RSAD2, OAS1, IFIT1, ISG12(A), BST2, IFI44, XAF1, and RTP4). The differences between upper and lower trachea could be due to differences in cell composition as especially ciliated cells and submucosal gland cells are more prevalent in the upper airway (39, 40). Another contributing factor could be the microenvironment, such as pH, oxygen levels, and the presence of commensal bacteria, which might differ significantly between upper and lower trachea, influencing the response to infection (41, 42). In lower trachea, IFI6 was one of the highest induced antiviral factors at both the protein and RNA level after either infections, indicating an important function in the antiviral immune response regardless of host adaptation level and replication capacity of the infecting strain. To our knowledge, IFI6 expression in tracheal tissue after IAV infection has never been described in a mammalian host before, contrary to other ISGs, such as ISG15, MX1, MX2, OAS1 and EIF2AK2 (PKR) (20, 43, 44), and cytokines/chemokines, such as IL6, IL8, IL1B, and CXCL10 (16, 44), which were also identified in this study. In a human respiratory epithelial cell line, A549, *IFI6* was, in agreement with the present study, identified to be important and highly upregulated during infection with several different IAV H1N1 strains as well as other subtypes, including avian H5N2, H5N3, and H9N2 (45). The role of IFI6 has been described in relation to other viral pathogens, e.g. inhibition of hepatitis B virus transcription and replication (46) and hepatitis C virus entry and replication (47). In the case of IAV and SARS-CoV-2, IFI6 has been demonstrated to dampen the innate immune response by negatively affecting RIG-I activation (48). Thus, IFI6 might contribute to suppression of the antiviral immune response after infection with both swH1N1 and huH1N1. Though, IFI6 expression was higher after infection with huH1N1, especially at the protein level (∼50%), which might contribute substantially to the observed lower antiviral response. Research in IAV infected tracheal tissue from a mammalian host has mainly been focusing on T cell infiltration (15–17), but here we demonstrate induction of the innate immune response via interferons and several central interferon stimulated genes and proteins in tracheal tissue after infection with either swH1N1 or huH1N1.

Despite the similarities between the antiviral responses after infection with the two IAV strains, important differences in activation of the innate pathways were also observed. The JAK-STAT signaling pathway has an essential role in regulation of the antiviral and inflammatory response during viral infections and has not been described previously in any tracheal tissue *in vivo*. In the present study, mRNA coding for cytokines and cytokine receptors involved in the JAK-STAT pathway, including *IL19, IL20, IL21R*, and *IL22RA2*, were highly expressed in lower trachea during host-adapted swH1N1 infection compared to non-adapted huH1N1 infection. However, the STAT1 protein was at lower abundance at the sampling time, highlighting the intricate temporal dynamics between gene expression and protein turnover in the JAK-STAT pathway and the importance of a multi-omics approach, which enable the examination of current proteins as well as the prediction of (potential) future proteins. The protein EP300, a transcriptional coactivator of STAT1 (49), was significantly increased during swH1N1 infection compared to huH1N1 infection, but as STAT1 was suppressed, the EP300 protein might be activating other transcription factors, e.g. IRF (50, 51) and NF-kb (52) contributing to the high expression of cytokines and chemokines observed after infection with swH1N1. Differences in virus sequences might also contribute to the observed variations in the antiviral responses after the two infections. We found that NS1 was the viral protein with the third lowest amino acid identity between the two inoculum strains, only surpassed by the two hyper variable surface proteins (HA and NA). Rajsbaum and colleges (53) demonstrated that NS1 inhibits the RIG-I pathway in a species-specific manner, as only NS1 proteins from human-adapted IAV strains (H1N1 and H3N2) showed binding to the RIG-I activating ubiquitin ligase RNF135 (Riplet) in a human cell line (53). Furthermore, it has been shown that a glutamic acid (E) in position 55 of the NS1 residue instead of a lysine (K) enhances viral replication and interferon suppression (54) and swH1N1 has an glutamic acid in position 55 (E55) in its NS1 sequence, while huH1N1 has a lysine (K55) (**Supplementary Figure S4**). NS1 binding to CPSF4/CPSF30, which is important for processing host pre-mRNA, might also contribute to the observed differences in antiviral responses. Here, an aspartic acid (D) in residue 125 is important for interaction, which is present in the huH1N1 NS1 sequence, but not in the swH1N1 NS1 sequence (**Supplementary Figure S4**). The huH1N1 could potentially have increased binding affinity for CPSF4, which could contribute to the observed reduced host immune response. Loss of CPSF4 binding does not seem to interfere with IFN inhibition, but it is correlated with a greater induction of pro-inflammatory cytokines and subsequent a greater pathogenicity (54), which is what we have observed after infection with swH1N1. The observed variations were not due to an inability of huH1N1 to replicate as infectious virus was demonstrated in the lungs after both infections. Though, we detected a tendency of a faster clearance of huH1N1, as fewer huH1N1-infected pigs had detectable infectious virus in the lungs and no detectable viral RNA in nasal swab samples at day 7 and day 10, where RNA was detected at the same time points in one or more animals after infection with swH1N1 (25). Thus, despite a lower production of differentially expressed genes upon infection with huH1N1, the immune response was sufficient to control the infection with induction of important antiviral immune factors, such as the OAS family, the IFIT family, MX, ISG15, IFI6, RSAD2, BST2 etc. The infection with the swH1N1 virus induced a high number of antiviral genes, but the virus’ ability to still replicate efficiently might be due to a better capability to evade parts of the immune response, potentially through NS1 as proposed and/or a more abundant HA attachment (55, 56).

Histological findings of immune cell infiltration in tracheal tissues after infection with swH1N1 was in line with induction of cytokines and chemotactic factors (*AMCF-II, CXCL8/IL8, CXCL2, CXCL10*, and *CCL20*) as well as receptors (*CCR1* and *CXCR2*) in lower trachea after this infection. Immune cell infiltration in the tracheal tissues was only seen to a minor degree in the upper trachea after infection with huH1N1. T cell infiltration and the presence of NK cells and neutrophils has been described in mice tracheal tissues infected with mouse-adapted H1N1 and H3N2 IAV (15, 16) and mononuclear cell infiltration is observed in tracheal tissue from fatal human cases of H1N1 IAV infection (57), but linking the tracheal histopathological differences with inflammatory immune responses after viral infection and between host-adapted IAV and non-adapted IAV has not been described in a large animal model before. The higher degree of single cell necrosis observed in tracheal tissue after infection with swH1N1 (**Figure 5**) could be linked to inflammation and apoptosis, driven by the elevated transcription of pro-inflammatory cytokines (*IL1A, IL1B, IL6, IL19, IL20*, and *IL27*), inflammatory related genes from the S100 family as well as pro-apoptotic genes and proteins (CAPN14, USP18, XAF1, Caspase-13/CASP4, ISG12, and ANXA5) in lower trachea. In agreement with the histological findings, no cytokines or chemokines were induced in the upper and lower trachea after infection with huH1N1 (**Supplementary Table S3**, **S5**). Only a single pro-apoptotic protein, ANXA5, was higher in abundance after infection with huH1N1 in lower trachea, and the anti-apoptotic DFFA was lower in abundance in upper trachea (**Supplementary Table S3**). A higher degree of apoptosis could be induced by the host-adapted IAV to increase the release of progeny viruses and spread of these to neighboring cells, but it could also be an important response of the innate immune system in order to kill infected cells (58). Anti-apoptotic factors including serine protease inhibitors (SERPINB10, SERPINA3–8 (Serpin domain-containing protein, LOC106504547), and SERPIN11A) and heat shock proteins (*HSP70*.2 and *HSPA6*), were only found after infection with swH1N1 compared to control. None of the serine proteases have been described in relation to viral infections before, but together with the heat shock proteins and the pro-apoptotic factors they might play an important role in a tight regulation of IAV induced apoptosis in its native host. This balance between an efficient infection with induction of a higher viral load compared to the non-host-adapted IAV and the ability to control pathogenicity/cell death might be acquired during host adaptation and would be an important factor during zoonotic events.

The cytoskeleton is actively involved in virus entry, assembly, and release and both viral proteins (HA, NP, and M1) and the eight viral ribonucleoproteins interact with cytoskeletal elements (59–63). Genes (*NEB, MYOM2, MYOZ1*, and *MYBPC1*) coding for cytoskeleton elements were downregulated, while other genes (*KRT13, KRT17, KRT6A*, and *BFSP1*) were upregulated along with higher abundance of the proteins (PLEC, KRT2, and PLXNA4) after infection with swH1N1 compared to both control pigs and pigs infected with huH1N1. In addition, transcripts coding for S100 proteins (S100A2, S100A3, and S100A8) were upregulated after swH1N1 infection, where S100A2 and S100A8 are involved in regulation of cytoskeleton dynamics (64–67). Further supporting the important role of the cytoskeleton during IAV infection, global proteomics showed that S100A1, which has an inhibitory effect on microtubule assembly (68, 69), was less abundant following exposure to swH1N1 as opposed to huH1N1. Regulation of cytoskeleton movement may be advantageous for IAV in order to decrease virus removal from the infected epithelial cells by mucociliary clearance, and at the same time optimize cytoskeleton movement within the cell for viral entry, assembly, and release. Contrasting findings have been reported in mouse trachea after IAV infection as a decrease in mucociliary velocity has been demonstrated *in vivo (* 19) and an increase of ciliary activity was shown *ex vivo (* 18). Only three proteins (GSN, PLEC, and STRIP1) and one transcript (*MYOM3*) related to the cytoskeleton organization and dynamics were regulated in the lower trachea after infection with huH1N1. It might be speculated that viral adaptation to the cytoskeleton dynamics of the host cell is an important factor for cross-species transmission to ensure optimal pro-viral host cell metabolism and establishment in a new host.

Genes involved in pyrimidine metabolism (*RRM2*, *CTPS1*, *TK1, NME1, NME2*, and *UCK2*) were induced after infection with swH1N1 compared to huH1N1. RRM2 and TK1 is involved in DNA replication and repair (70, 71), while CTPS1 takes part in RNA synthesis (72, 73). NME1 and NME2 are involved in synthesis of nucleoside triphosphates of RNA and DNA, and UCK2 catalyses the phosphorylation of uridine and cytidine to uridine monophosphate (UMP) and cytidine monophosphate (CMP) (74). CTPS1 catalyzes the formation of cytidine triphosphate (CTP) from uridine triphosphate (UTP) (73), and is essential for influenza transcription as viral mRNA elongation will be stalled in the lack of CTP as has been described for both IAV and influenza B virus (75, 76). Furthermore, it has been suggested that CTPS1 inhibit IFN induction during SARS-CoV-2 infection (77). Thus, the swH1N1 manipulates host cell mechanisms to boost pyrimidine synthesis ensuring sufficient quantities of essential nucleotides for viral replication, which might be connected with the observed higher viral RNA loads, a higher degree of single cell necrosis, and immune factors linked to cell death. IAV replication has been described to be dependent on the pyrimidine biosynthesis (78, 79), but the exact mechanisms of how IAV regulates the pyrimidine pathway have to be investigated.

To conclude, a classical antiviral innate immune response against both host-adapted (swH1N1) and non-adapted (huH1N1) IAV infections was seen in the tracheal epithelium. Infection with the host-adapted swH1N1 virus induced a stronger immune response, with more activated innate immune genes, higher expression levels, single cell necrosis, immune cell infiltration, a higher viral load, and more infectious virus compared to infection with a non-adapted huH1N1 virus in the lower trachea. Furthermore, infection with the swH1N1 resulted in regulation of genes and proteins involved in cytoskeleton dynamics and organization, pyrimidine metabolism, apoptosis, and the JAK-STAT pathway. This suggests that with adaptation swH1N1 IAV has evolved to favor viral infectivity and replication. Using a multi-omics approach many different host factors were investigated during IAV infection and based on our findings, it could be speculated that the likelihood of a cross-species infection and establishment in a new host might be connected to the virus’ ability to interact and regulate the host cell environment to improve the chance of a successful and transmittable infection.

## Data Availability

The datasets presented in this study can be found in online repositories. The names of the repository/repositories and accession number(s) can be found below: GSE268254 (GEO) and PXD052229 (ProteomeXchange).
