# Supplementary material for: Multi-omics analysis reveals the impact of influenza a virus host adaptation on immune signatures in pig tracheal tissue
Source: Front Immunol. 2024 Aug 16;15:1432743. doi: 10.3389/fimmu.2024.1432743 (PMC11378526; doi:10.3389/fimmu.2024.1432743)
Supplement: Supplementary file 1 [file Image1.pdf]

Supplementary Figures

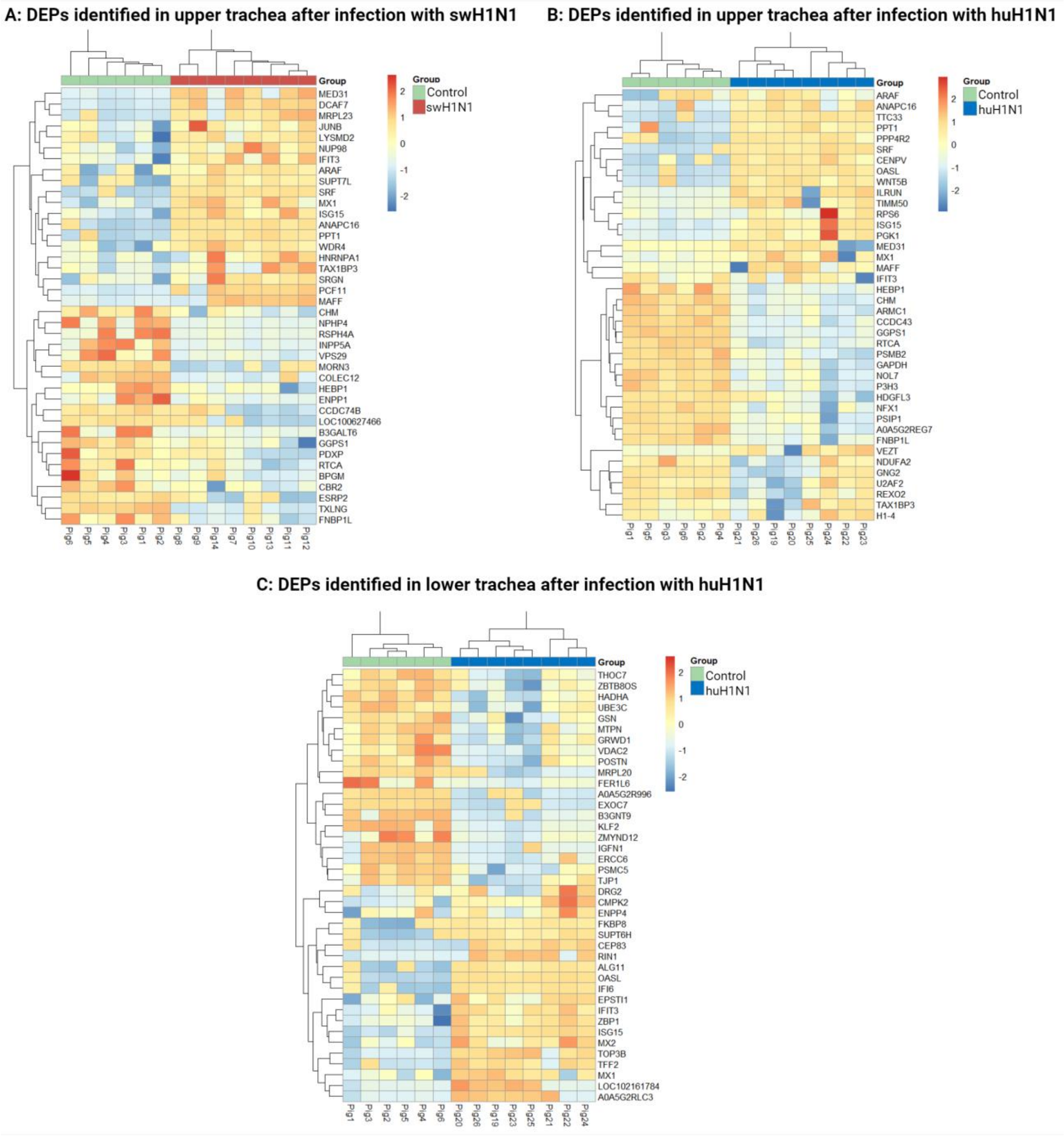

Supplementary Figure S1: Heatmaps of DEPs in upper and lower trachea after either IAV infections. **(A)** Top 20 significantly higher or lower abundant proteins (based on padj) in upper trachea after infection with the swH1N1 (red) compared to control (green). **(B)** Top 20 significantly higher or lower abundant proteins (based on padj) in upper trachea after infection with the huH1N1 (blue) compared to control (green). **(C)** Top 20 significantly higher or lower abundant proteins (based on padj) in lower trachea after infection with the huH1N1 (blue) compared to control (green). The colour key from blue to red indicates low to high protein expression, respectively.

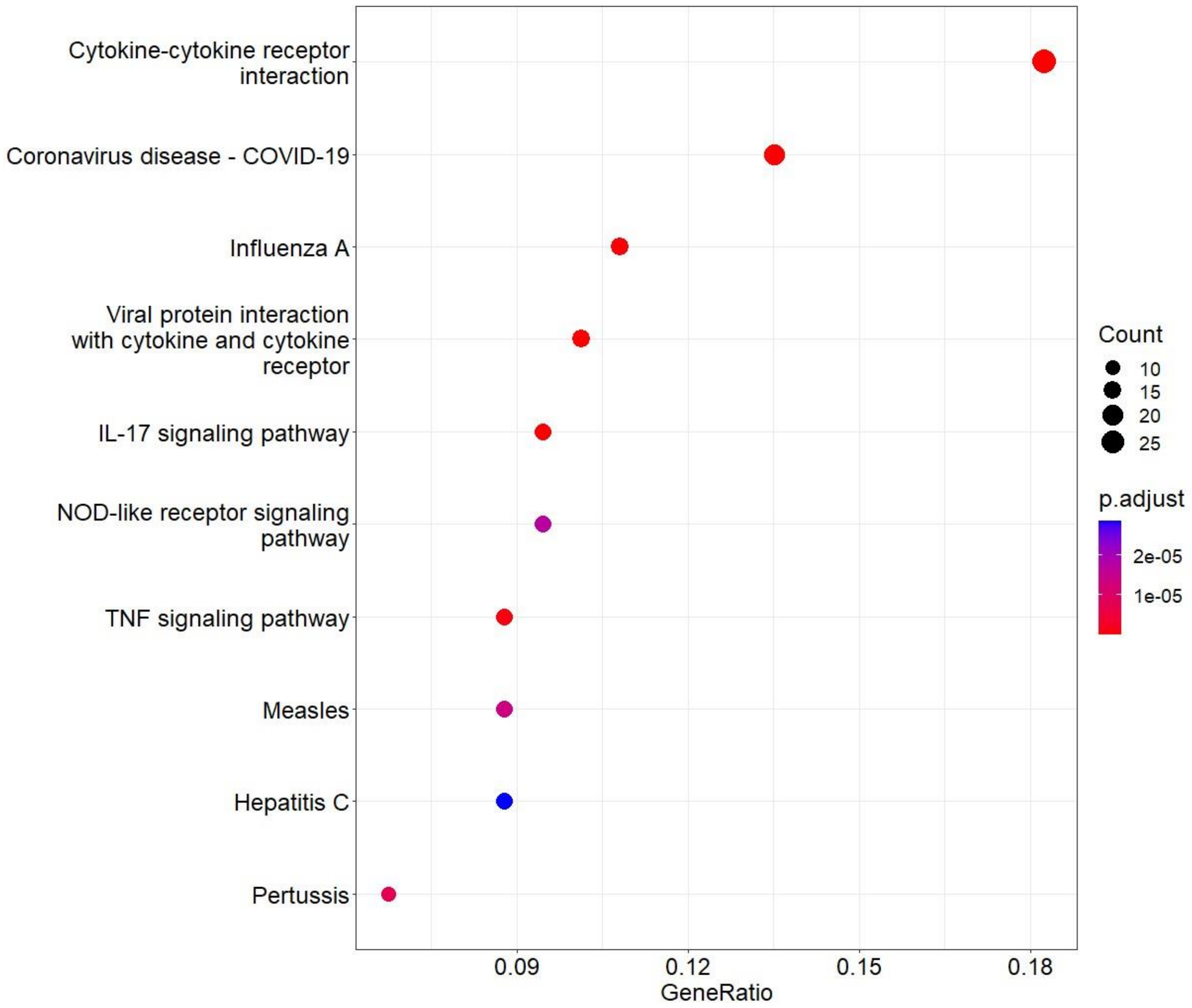

Supplementary Figure S2: KEGG enrichment analysis of DEGs and DEPs in lower trachea after infection with swH1N1 compared to control. The bubble diagram indicates the ratio of enriched DEGs to the total number of identified genes in a certain pathway. Circles indicate the number of genes in the corresponding pathway, and color depicts the adjusted p-value.

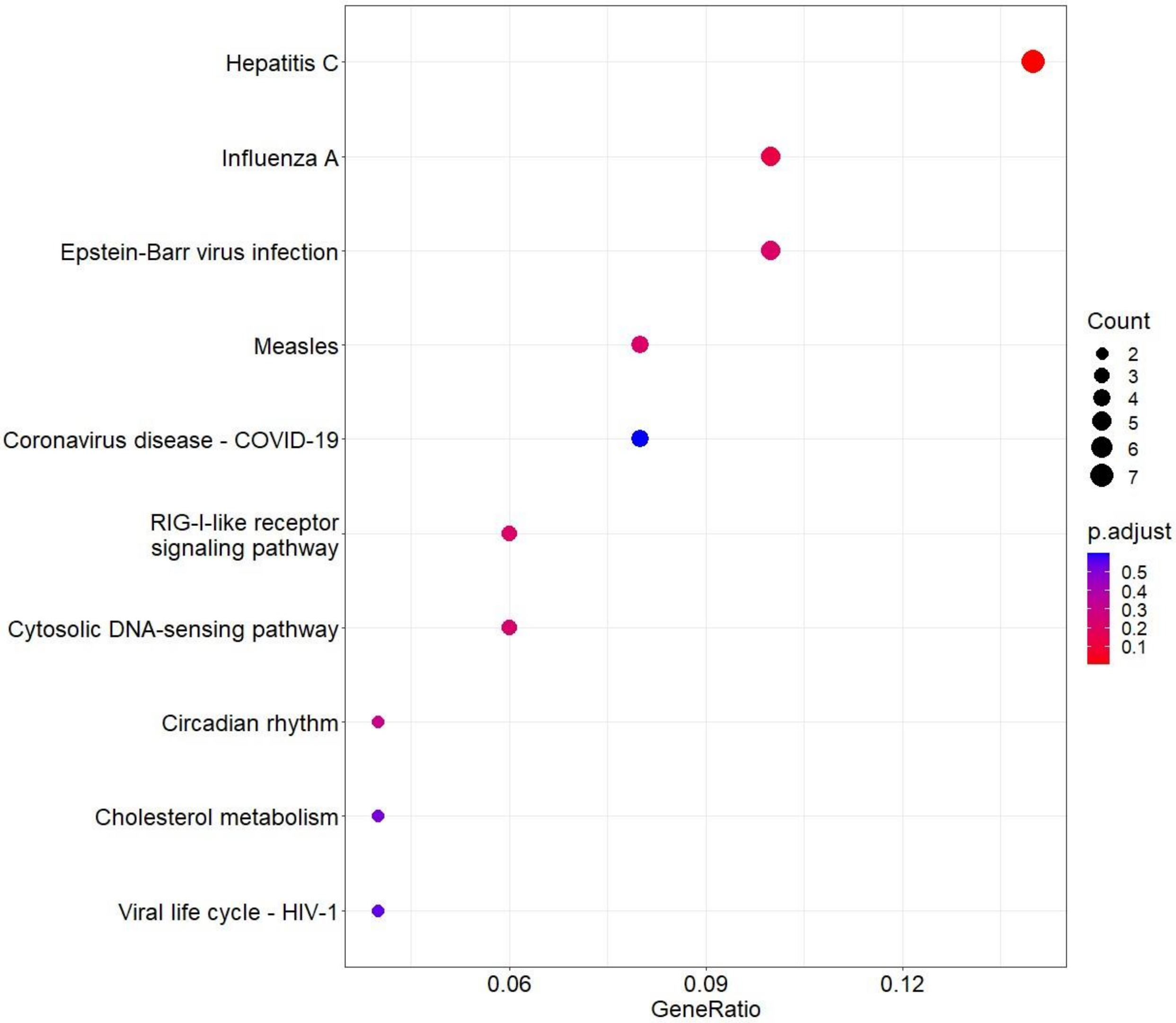

Supplementary Figure S3: KEGG enrichment analysis of DEGs and DEPs in lower trachea after infection with huH1N1 compared to control. The bubble diagram indicates the ratio of enriched DEGs to the total number of identified genes in a certain pathway. Circles indicate the number of genes in the corresponding pathway, and color depicts the adjusted p-value.

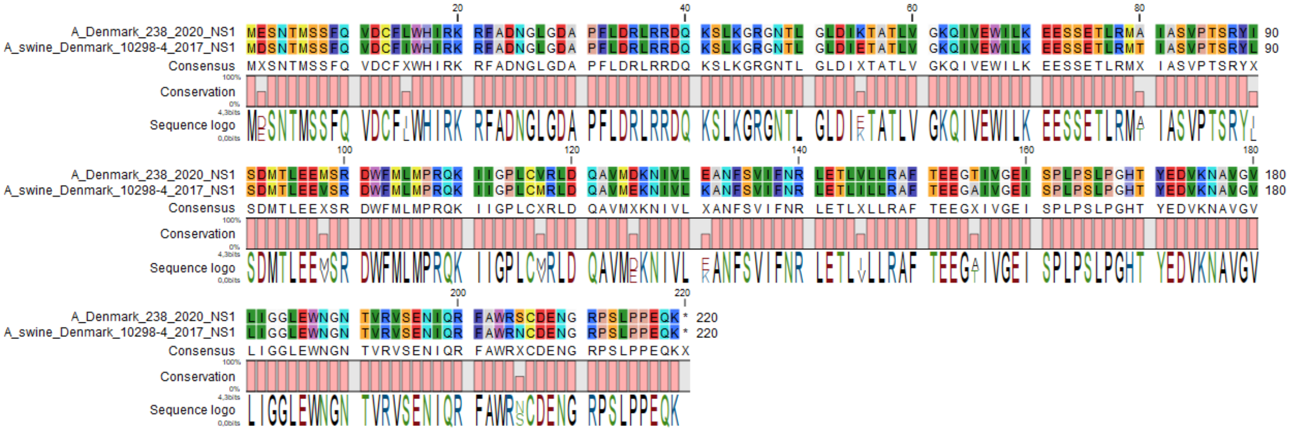

Supplementary Figure S4: Sequence alignment of the amino acid sequences of the NS1 gene of the two IAV strains included in the study. The alignment was performed using CLC Main Workbench Version 22.0.2 (QIAGEN).
